# Evaluation of the Taxonomic Status of Lesser Egyptian Jerboa, *Jaculus jaculus*: First Description of New Phylogroups in Tunisia

**DOI:** 10.3390/ani12060758

**Published:** 2022-03-17

**Authors:** Wissem Ghawar, Melek Chaouch, Souha Ben Abderrazak, Mohammed Ali Snoussi, Sadok Salem, Said Chouchen, Amor Bouaoun, Afif Ben Salah, Jihene Bettaieb

**Affiliations:** 1 Department of Medical Epidemiology, Institut Pasteur de Tunis, Tunis 1002, Tunisia; mohamedali.snoussi@pasteur.tn (M.A.S.); sadok-salem@live.fr (S.S.); afif.bensalah@pasteur.tn (A.B.S.); jihene.bettaieb@pasteur.tn (J.B.); 2 Laboratory of Transmission, Control and Immunobiology of Infections (LR16IPT02), Institut Pasteur de Tunis, Tunis 1002, Tunisia; 3 Clinical Investigation Center (CIC), Institut Pasteur de Tunis, Tunis 1002, Tunisia; chouchensaid@gmail.com; 4 University Tunis El Manar, Campus Universitaire Farhat Hached, Tunis 1068, Tunisia; mcmelek@msn.com (M.C.); souha.benabderrazakklilib@pasteur.tn (S.B.A.); 5 Laboratory of Medical Parasitology, Biotechnology and Biomolecules (LR16IPT06), Institut Pasteur de Tunis, Tunis 1002, Tunisia; 6 Laboratory of Bioinformatics, Biomathematics and Biostatistics (LR16IPT09), Institut Pasteur de Tunis, Tunis 1002, Tunisia; 7 Health Regional Directorate of Tataouine, Tataouine 3263, Tunisia; amor.bouaoun@gmail.com; 8 Faculty of Medicine of Tunis, University Tunis El Manar, Tunis 1068, Tunisia; 9 Department of Family and Community Medicine, College of Medicine and Medical Sciences (CMMS), Arabian Gulf University (AGU), Manama 329, Bahrain

**Keywords:** *Jaculus hirtipes*, *Jaculus jaculus*, rodent, taxonomy, Tunisia

## Abstract

**Simple Summary:**

Rodents systematically represent a major issue owing to their biodiversity assessment. These micromammals exhibit phenotypic and genetic diversity and can be found in different ecosystems. The current study was undertaken to examine the morphometric and genetic patterns recently adapted by *Jaculus* (*J*.) *jaculus* in Tunisia. Moreover, we investigated micro-geographic clustering according to the species and/or the biotope ecosystem. Forty-six rodents were captured and analyzed for body measurements. At the genetic level, we confirmed the presence of two species, *J. jaculus* and *J. hirtipes*, but we found that each of these rodent species harbored two phylogroups that were genetically distant without any geographical and/or environmental structuring.

**Abstract:**

The taxonomy of the Lesser Egyptian jerboa, *Jaculus* (*J*.) *jaculus* (Dipodinae subfamily), was recently reevaluated, and the taxonomic status was defined by the presence of two cryptic species, *J*. *jaculus* (Linnaeus 1758) and *J*. *hirtipes* (Lichtenstein, 1823), with a higher genetic divergence in the sympatric North African populations than in other studied parapatric populations. Using phylogenetic analysis of the cytochrome b (*Cytb*) gene from 46 specimens, we confirmed the new status in Tunisia; rodents were collected from two different biotopes belonging to the same locality at the ecological level (mountainous vs. Saharan) in the south of the country. The study of the eye lens weight of these specimens allowed the definition of a cutoff value (58.5 g), categorizing juveniles from adults. Moreover, this study confirmed the phylotaxonomic status of *J*. *jaculus* in Tunisia, as recently illustrated, into two distinct species, *J*. *jaculus* and *J*. *hirtipes*, and recorded for the first time the presence of two phylogroups among each of these rodent species. The lack of clear micro-geographical structure and biotope specificity between the two rodent species and their phylogroups was also highlighted.

## 1. Introduction

The Lesser Egyptian Jerboa *Jaculus* (*J*.) *jaculus* (Linnaeus, 1758) is endemic to North Africa, northeastern Africa, the Arabian Peninsula, and southwestern Asia [1,2]. This wide geographical distribution and frequent shifts in climate, in addition to the morphological and ecological polymorphisms observed within and among places, allows reclassification of this species into two independent lineages [3] with a sympatric distribution in North and North-West Africa [4]. Several studies have focused intensively on phenotypic and genetic variability analysis of Lesser Egyptian Jerboa specimens from the Middle East and North Africa [1,4,5,6,7,8,9,10]. It has been proposed that these lineages may be reproductively isolated, forming two closely related cryptic species [1,4,5,6,9,10,11,12,13]. Some of the later studies provided evidence of the existence of three distinct clades, which are well discriminated against using molecular tools based on nuclear and mitochondrial DNA [8,10]. Two of these clades have been reported to be widely sympatric across North Africa, and the genetic difference between them exceeds the average recorded between rodent species [4,8]. The third clade, spreading in the Middle East and Arabian Peninsula, has not been reported in Tunisia and appears to be allopatric [4,8].

In Tunisia, the taxonomy of *J*. *jaculus* is controversial [1,5]. The clades have sometimes been related to two well-differentiated species, *J*. *jaculus* and *J*. *deserti*, although this has not been recognized in recent taxonomic updates [8]. Because of these nomenclature-related ambiguities, several authors have conservatively used neutral denominations (Clades A and B or Clades I and II) for these forms [1,10]. Recent reports [4,10] have revealed the presence of two distinct species, *J*. *jaculus* (Linnaeus, 1758) and *J*. *hirtipes* (Lichtenstein, 1823). The previously described third clade was integrated into the *J. hirtipes* clade [4]. 

The present study aimed to compare the age structure, morphological measurements, and molecular variability based on the cytochrome b (*Cytb*) gene of the *Jaculus* genus occurring in the Tataouine governorate in South Tunisia. Moreover, their genetic divergence at a micro-geographical scale into two different ecological systems (Saharan vs. mountainous) was also investigated. Such information is needed to evaluate biotope preferences, the relative importance of sympatry, and the genetic diversity between and among rodent species in different ecosystems at a micro-geographical scale. 

## 2. Materials and Methods

### 2.1. Ethical Statement 

All animal experiments complied with institutional, national, and international guidelines. The study and rodent handling protocols were approved by the Biomedical Ethics Committee of the Institut Pasteur de Tunis (document reference: 2016/13E/I/CIC).

### 2.2. Study Sites and Sampling

**Figure 1:**
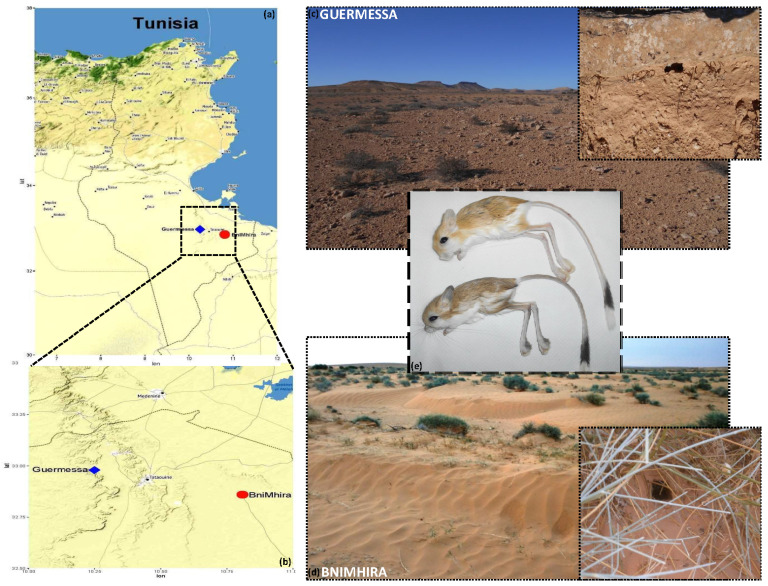
Study sites of *Jaculus* species. (**a**) Image of the location of the study area (determined by icons) at Tataouine Governorate. (**b**) Zoom into the locations of the two study sites highlighting the mountainous relief of Guermessa and Saharan relief of BniMhira. (**c**) The study site of Guermessa emphasizes rodents’ burrows. (**d**) The study site of BniMhira emphasizes rodents’ burrows. (**e**) The Tunisian *Jaculus* species: *Jaculus jaculus* (the one at the top of the image) and *Jaculus hirtipes* (the one at the bottom of the image), showing morphological and color differentiation between the two species.

Rodents were trapped from two study sites in the Tataouine governorate in the South of Tunisia: (i) BniMhira (average altitude 231 m; 32°87′62.52″ N 10°79′09.95″ E), a sandy Saharan biotope; and (ii) Guermessa (average altitude 297 m; 32°98′75.70″ N 10°26′88.71″ E), a rocky-steppe region located beside the mountains. Biotopes were mapped based on global positioning system (GPS) coordinates using R software v. 4.1.3 (Figure 1).

*Jaculus* spp. were collected at night between May 2017 and June 2018 using butterfly nets. Five field trips (one week each) were conducted: three between May and June 2017 and two in May and June 2018 with an equal alternation between the two zones. The trapped rodents were then transported to a local field laboratory for examination. 

### 2.3. Morphometric Measurements 

Following sex determination by visual inspection of the external genitalia [14,15], rodents were weighed, and standard morphometric measurements were taken: head and body length (HBL), tail length (TL), ear length (EL), and hindfoot length (HFL), using a 1-mm graduated scale, as previously described [14,16]. After morphological measurements, each rodent was euthanized using an intraperitoneal dose of ketamine (40–90 mg/kg) associated with xylazine (5–10 mg/kg) and sacrificed by cardiac exsanguination. Both eyes were removed and preserved in 10% formalin for at least 2 weeks. The lenses were extracted, dried for 2 h at 100 °C, and weighed on a pan balance with an accuracy of 0.1 mg [17]. 

### 2.4. Rodents Age Categorization

Animals were categorized as juveniles or adults using two methods: weight (≤35 g and >35 g, respectively) and HBL (≤104 mm and >104 mm, respectively), as previously described for *J*. *jaculus* [18]. The kappa coefficient of concordance was calculated to measure agreement between the two methods. The mean eye lens weight (ELW) for each rodent was used as the best indirect measure for age determination [19]. A cutoff value of ELW to classify rodents as juveniles or adults was determined using a receiver operating characteristic (ROC) curve constructed from concordant pairs obtained using both age categorization methods (weight and HBL). 

### 2.5. Statistical Analysis

Categorical variables were summarized as frequency counts and percentages, while quantitative variables were reported as the mean ± standard deviation (SD) or the median with an interquartile range. Comparisons between group means were performed using Student’s *t*-test, analysis of variance (ANOVA), or the Kruskal–Wallis test; a chi-square test was used for comparison between group percentages. Statistical significance was set at a value of *p* ≤ 0.05 and interpreted using the Bonferroni correction method for multiple comparisons. 

### 2.6. DNA Extraction and Polymerase Chain Reaction (PCR) Conditions

DNA was extracted from 25 to 50 mg of rodent liver using the mammalian tissue protocol from the Wizard^®^ Genomic DNA Purification Kit (Promega, Madison, WI, USA). To identify rodent species, amplification of the rodent mitochondrial *Cytb* gene was performed using PCR. The L14723 forward (5′-ACCAATGACATGAAAAATCATGGTT-3′) and H15915 reverse (5′-TTCCATTTCTGGTTTACAAGAC-3′) primers were used to amplify a ~1100 bp segment [1]. PCR reactions were conducted in a 25 μL volume/reaction, containing 10× buffer supplemented with 1.5 mM MgCl_2_ (Qiagen), 0.1 mM dNTPs, 0.5 U of Taq DNA polymerase, and 1 µM of each primer. Reactions were performed in an Applied Biosystems (Foster City, CA, USA) GeneAmp PCR System 2700 using the following cycling conditions: an initial denaturation step at 94 °C for 3 min; 37 cycles of 94 °C for 30 s, 56 ° C for 1 min, and 72 °C for 1 min; and a final elongation step at 72 °C for 10 min [1]. The amplicons were electrophoresed on 1% agarose gel, stained with ethidium bromide, and visualized under UV light.

### 2.7. DNA Sequencing

DNA fragments obtained after PCR were purified using a QIAquick PCR purification kit (QIAGEN, Hilden, Germany) according to the manufacturer’s instructions. DNA sequences were determined using a conventional Big Dye Terminator Cycle Sequencing Ready Reaction Kit (Perkin Elmer, Applied Biosystems) and an ABI373 Automated DNA Sequencer. The obtained sequences were edited using ChromasPro software (Technelysium Pty Ltd., South Brisbane, Australia). For the homology search, sequences were submitted to the National Center for Biotechnology Information (NCBI) server using a basic local alignment search tool (BLAST. Available online: http://blast.ncbi.nlm.nih.gov/ (accessed on 21 June 2021). 

### 2.8. Maximum Likelihood Phylogenetic Analyses

The phylogenetic relationship study was based on the alignment of the generated *Cytb* sequences and other rodent reference sequences using Clustal software (University College Dublin, Dublin, Ireland) [20]. The output was edited, and regions of unambiguously aligned sequences were retained for the final analysis. The new MEGA software version (Pennsylvania State University, State College, PA, USA) [21] was used to determine the best model using the Akaike information criterion. A phylogenetic tree was generated using the HKY+G model and performed with the maximum likelihood (ML) method available in SeaView4 (Lyon 1 University, Lyon, France) [22]. Using the optimization options, 1000 bootstrap replicates were performed. *J. orientalis* (JN652641) and *Psammomys vexillaris* (MF687143) sequences were used as outgroups. In addition to the sequences retrieved in the present study, all Tunisian sequences derived from Lesser Egyptian Jerboa were used for *Cytb* phylogenetic analysis. The accession numbers of the sequences are listed in Table S1. Moreover, all GPS coordinates of these sequences were used to generate a map illustrating the geographical distribution of each rodent species in Tunisia. R software (R Foundation for Statistical Computing, Vienna, Austria) was used to create a map with a base layer obtained from https://rdrr.io/cran/ggmap/man/get_stamenmap.html (accessed on 5 February 2022).

### 2.9. Genetic Diversity and Polymorphism Analysis

Standard genetic diversity indices were as follows: number of haplotypes (H), haplotype diversity (Hd), polymorphic sites (S), nucleotide diversity (π), average number of nucleotide differences (K) [23] and neutrality tests: Tajima’s D [24] and Fu’s F-statistics [25] (without out-group), and their standard deviations were estimated using DnaSP version 6.12.03 (Julio Rozas & Universitat de Barcelona, Barcelona, Spain) [26].

### 2.10. Genetic Differentiation and Gene Flow among Rodent’s Species and Their Phylogroups

The Ks *, Kst *, Z *, *Snn*, and *Fst* values [27,28], which determined genetic differentiation on the *Cytb* nucleotide sequences among the two species (*J. jaculus* vs. *J. hirtipes*) and the four phylogroups (two phylogroups identified among each rodent species), were calculated using DnaSP [26]. Kst * will be near zero if there is no genetic differentiation (null hypothesis) [29]. A smaller Z * indicates smaller genetic differentiation among populations [28]. The value of *Snn* describes a range of the same population (value of 0.5) (null hypothesis) to distinctly differentiate the population (value of 1) [27]. The null hypothesis in these tests was rejected by a significant *p*-value [27,28,29]. *Fst* ranges from exactly the same population (value of 0) to fully distinct populations (value of 1) [28,29]. *Fst* > 0.25 in most cases indicates a large gene flow and genetic differentiation in the tested populations [30].

## 3. Results

### 3.1. Rodents Sampling and Morphological Measurements 

Forty-six rodents were captured and morphologically identified as belonging to the genus *Jaculus*. Thirty (*n* = 30; 65.2%) were captured in BniMhira and 16 (34.8%) in Guermessa. Among the collected rodents, there were 10 males (21.7%) and 36 females (78.3%), of which four harbored one to four embryos.

The weight, ELW, and morphological characteristics of the captured rodents are presented in Table 1. Among all captured rodents, no significant differences were detected according to sex (*p* values = Weight: 0.731; HBL: 0.791; TL: 0.873; EL: 0.337; HFL: 0.579; ELW: 0.207) or location (*p* values = Weight: 0.393; HBL: 0.73; TL: 0.428; EL: 0.075; HFL: 0.414; ELW: 0.574), despite the presence of minor visual morphological differences, such as the type of the bicolor tail brush: an incomplete black band on the ventral side of the tail banner with an enlarged and flattened white-tipped bushy tail vs. a complete black band on the tail banner with a smothered white-tipped bushy tail resembling a narrow bottle brush (Figure 1). 

Morphological comparisons between the two rodent species (identified by the phylogenetic study below) revealed a significant difference for only one variable (Table 2). *J. hirtipes* had significantly longer ears compared to *J. jaculus* (*p* = < 10^−3^). According to sex, *J. hirtipes* males had longer ears than females (23.33 ± 0.816 vs. 21.79 ± 1.051; *p* = 0.005 < *p* = 0.0083), while no significance for all parameters was recorded between *J. jaculus* gender groups and for both rodent species according to biotopes.

### 3.2. Rodents Age Categorization

Agreement between both previously described age categorization methods identified 11 juveniles and 28 adults, resulting in a moderate kappa coefficient of concordance (0.65). Furthermore, the mean ELW was significant (*p* ≤ 0.05) for both methods. Based on the concordant specimens between both age categorization methods, the ROC curve permitted the determination of an ELW value of 58.5 as the cutoff (sensibility 96.4% and specificity 90.9%) between juveniles and adults. This cut-off permitted reclassification of all specimens into juveniles and adults (Table 3). Three morphometric measurements, weight, HBL, and TL, were statistically significant (*p* ≤ 10^−3^) in juveniles and adults categorized using the ELW cutoff. 

### 3.3. Phylogenetic Analyses

**Figure 2:**
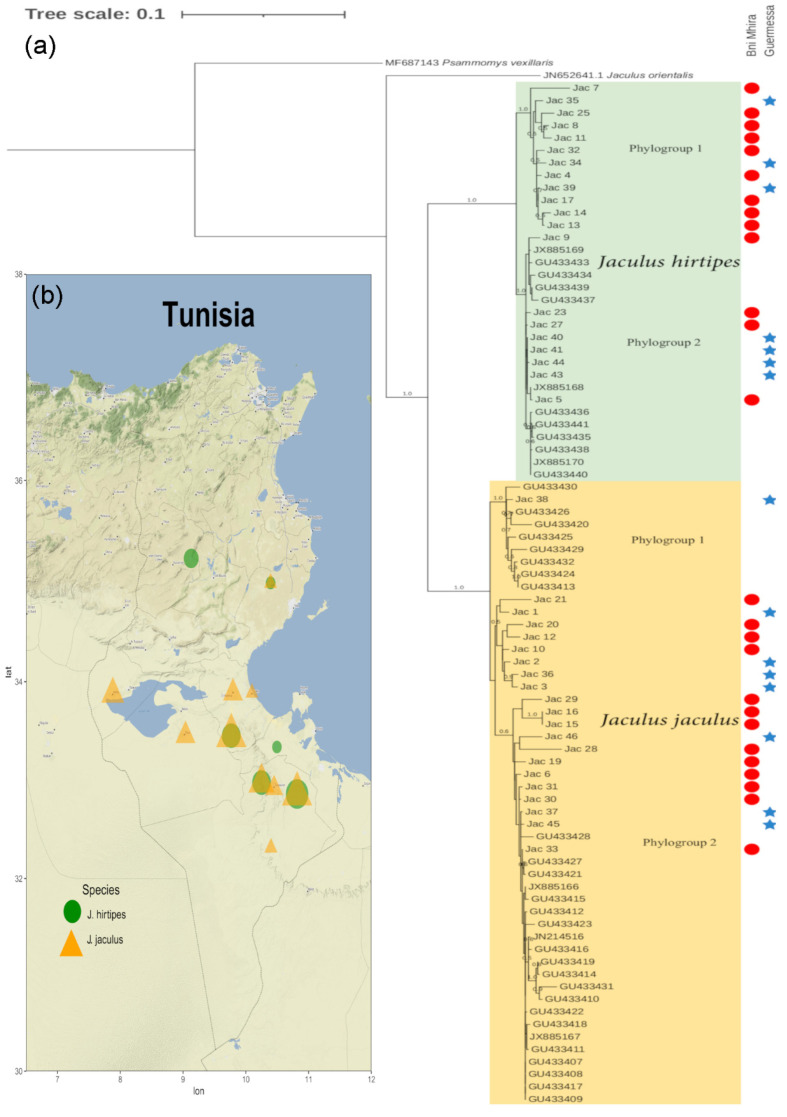
Phylogenetic relationship of new specimens and the Tunisian reference samples of *J. jaculus* and *J. hirtipes*, and their geographical distribution. (**a**): Phylogenetic tree based on maximum likelihood showing the relationship among Tunisian sequences for cytochrome b (*Cytb*) gene. Values on branches indicate bootstrap values. *Jaculus orientalis* and *Psammomys vexillaris* were used as outgroups. (**b**): Geographical locations of all Tunisian specimens used in the study assigned by rodent species. Yellow triangles and green circles correspond to *J. hirtipes* and *J. jaculus*, respectively. The size of the symbol is relative to the sample number at that location.

A total of 41 *Cytb* sequences were successfully amplified. Forty-two reference sequences retrieved from GenBank were used in this study. Aligned sequences of 953 bp were obtained after trimming ambiguous sites. Phylogenetic analysis of different Tunisian samples, performed using the ML method, showed a clear topology, with two diverging and strongly supported clades (bootstrap = 1). The phylogenetic tree highlighted the existence of two well-supported species clades corresponding to *J*. *jaculus* and *J*. *hirtipes*, as was recently identified. Twenty-one of the new sequences were recognized as belonging to *J*. *jaculus*, while the other 20 sequences belonged to *J*. *hirtipes*. No geographical or biotope differentiation was detected between the clades in our samples. Both rodent species were found in the two studied biotopes (Figure 2).

Interestingly, two separate clusters (phylogroups) within each identified rodent species clade were observed with a high bootstrap value (bootstrap = 1) among the *J. hirtipes* clade. For the *J*. *jaculus* clade, the first cluster was highly supported (bootstrap = 1), while the second cluster was poorly supported (bootstrap < 0.5) and subdivided into two groups that were also poorly supported (bootstrap = 0.5–0.6) (Figure 2). Among *J. hirtipes*, the first phylogroup was strictly reserved for 12 sequences of specimens from Tataouine, with a mixed biotope origin: three from Guermessa and nine from BniMhira. The second phylogroup gathered the rest of the Tunisian sequences belonging to this rodent species from different localities (Gabes, Medenine, Kasserine, Sfax, and Tataouine) (Figure 2). *J. jaculus* was also divided into two phylogroups, the first containing only one of the sequences from Guermessa and eight Tunisian reference sequences from two other localities in addition to Tataouine: Gabes and Tozeur in the south of the country, and the second phylogroup harboring the rest of the sequences from the south and the center of the country (Gabes, Tozeur, Sfax, and Tataouine). The latter was split into two groups, it was although poorly supported. Eight of the sequences (four from each biotope) were grouped (bootstrap = 0.5), while 12 sequences were grouped with the rest of the Tunisian reference sequences (bootstrap = 0.6).

A map showing the geographical distribution of both rodent species in Tunisia (Figure 2b), using the GPS coordinates of the sequences used in the phylogenetic analysis, confirmed their presence in two biotopes at Tataouine for the first time, in addition to their previously described data in Gabes and Sfax.

### 3.4. Genetic Diversity and Polymorphism Analysis

All standard diversity indices for the 41 Tunisian *Jaculus Cytb* sequences from the present study and for all Tunisian sequences found in the NCBI database are summarized in Table 4. Indexes according to capture biotopes and phylogenetic species identification were also calculated (Table 4). 

For our dataset, 38 haplotypes were identified: 20 from *J. jaculus* and 18 from *J. hirtipes*. No haplotype was shared between either rodent species or between specimens belonging to both biotopes. Each specimen recovered a haplotype, except for three specimens (Jac 40, Jac 41, and Jac 43) gathered in one haplotype from *J*. *hirtipes* and two specimens (Jac 15 and Jac 16) from *J*. *jaculus*. The polymorphic sites (S) were observed to be higher in *J. jaculus*, either in new sequences or in the entire dataset. Interestingly, it almost doubled between phylogroups for both rodent species (Table 4). The average number of nucleotide differences (K) was similar (maximum difference of two nucleotides) between rodent species for both datasets. According to biotopes, *J. jaculus* from BniMhira presented a lower K value than those from Guermessa. This picture was inverted for *J. hirtipes* between the two biotopes. Moreover, K seems to be similar for *J. jaculus* phylogroups, whereas it was three times higher between *J. hirtipes* phylogroups. Maximum nucleotide diversity (*π*) values were obtained from the new sequences (Table 4). Between rodent species and according to biotopes, it was approximately 0.02. Between phylogroups, it was in the same range as *J. jaculus*, whereas the second phylogroup among *J. hirtipes* showed the lowest value (0.005). 

According to these genetic parameters, *J. jaculus* from Guermessa showed slightly higher polymorphism than those from BniMhira, whereas *J. hirtipes* from BniMhira were more polymorphic than those from Guermessa. This result reflects the biotope preference of each rodent species.

For neutral selection analysis (Table 4), non-significant negative values of Tajima’s D and Fu’s Fs statistical tests were observed for most groups and phylogroups. Exceptionally positive Tajima’s D and Fu’s Fs values were obtained for *J. hirtipes* collected from Guermessa. A significantly negative value of Tajima’s D was obtained for phylogroup 2 and the whole Tunisian *J. jaculus,* indicating their possible expansion.

### 3.5. Genetic Differentiation and Gene Flow among Rodent Species and Their Identified Groups and Phylogroups

All values of calculated tests (*K*s *, *K*st *, *Z* *, and *Snn*) obtained for *Cytb* gene comparisons among these respective rodent species (clades) and clusters (phylogroups) were significantly different (Table 5). This allows us to conclude that these clades (Tunisian *J. jaculus* vs. *J. hirtpes*) and clusters (*J. jaculus* phylogroup 1 vs. phylogroup 2, and *J. hirtipes* phylogroup 1 vs. phylogroup 2) are distinct from each other and deserve to be classified separately. 

The highest *Fst* value (0.812) was obtained from a comparison between the two rodent species clades (Table 5). In contrast, the *Fst* values between *J. jaculus* (0.4618) and *J. hirtipes* (0.6961) phylogroups allow us to consider a moderate and strong population subdivision.

## 4. Discussion

In Tunisia, several studies have been conducted on rodent species belonging to the genus *Jaculus* [1,4,5,6,7,8,10,13,31,32]. 

### 4.1. Rodent’s Morphometry

Body measurements of the captured rodents showed a wide range of body weights, HBLs, and TLs. These measurements are in agreement with those reported in Sudan [33], Algeria [34] and the Saharan and peri-Saharan regions of Africa [18]. There was little variation in the mean of each measurement, which could be explained by the number of specimens studied, the ratio of juveniles to adults collected, and local adaptation. Surprisingly, at the local level, all morphometric measurements were lower than those reported by Ben Falah et al. [1] except for the EL. This could be explained by the fact that this study collected specimens only from the south of the country, while their study also contained specimens from the center of the country. 

Significant differences in ear length parameter, highlighted in our study for the first time, can be an adaptation type related to each rodent species. Additionally, a continual morphological change in rodent species modifies the morphology and physiology of individual rodents in each population as a response to both the physical environment and interactions with other organisms, such as pathogens [35,36]. 

Our study showed that micro-geographical distribution related to the ecological specificities cannot affect the standard morphometric parameters among both species regardless that rodents captured in Guermessa were from a rocky mountainous biotope, whereas those captured in BniMhira were from a Saharan biotope. 

According to sex, significant variation in mean ear length measurements among *J. hirtipes* could be explained in part by the hazardous nature of the trapping method used in this study, which does not consider sex activity among this species of rodents, as well as the difference in the number of specimens belonging to each sex. The absence of a significant difference in body measurements according to sex or location among these rodent species has been previously noted and extended to craniodental measurements [1].

### 4.2. Rodent’s Age Categorization

The discordance between the two methods of age categorization previously described [18] indicates the need for a minor readjustment of those methods. The significant association between ELW and both age categorization methods could be helpful. The ELW cutoff (58.5 g) determined in this study is a useful parameter for differentiating between juveniles and adults among those specimens, especially knowing that ELW is the best indirect measure for age determination [19] and is statistically significant with both previously described methods for *J*. *jaculus* [18]. Furthermore, based on phylogenetic rodent species identification, the ELW cutoff was valid for all captured *Jaculus* species in this study.

### 4.3. Rodent Species Phylogenetic and Phylogroup Description 

Analysis of *Cytb* sequences associated with the morphological data from Tunisian *Jaculus* spp. demonstrated important genetic divergence and morphological differentiation between the two major groups of captured specimens. The distribution of specimens belonging to either described rodent species did not depend on geographical patterns, and individuals were found in the same geographical area. These two *Jaculus* species have broad and sympatric distributions in Tunisia, supporting the results obtained from the analysis of the *Cytb* sequences, karyotype, and craniodental characteristics [5,6,8,9,10]. These findings are in disagreement with the opinions of Ranck [3], Gharaibeh [37], and Pisano et al. [38], who claim that the second sympatric group is named *J. deserti*, and Corbet [39], who argued that there is only one species of Lesser Jerboa in North Africa. This study confirms the recent reevaluation of taxonomic status based on both mitochondrial and nuclear DNA of the Lesser Egyptian Jerboa *J*. *jaculus* from North Africa [4] and the Middle East, performed by Shenbrot et al. [10]. It confirms the presence of two species, *J*. *jaculus* (Linnaeus 1758) and *J*. *hirtipes* (Lichtenstein, 1823). Despite this sympatry, *J*. *jaculus* and *J*. *hirtipes* clades are separated with a high bootstrap value, similar to the dichotomy described in Tunisian rodents [1,8]. Furthermore, the absence of intraspecific geographic structure observed at this level for the studied Tunisian rodents can be explained by the fact that Tunisia is considered one of the contact zones harboring two described highly differentiated lineages within the *Jaculus* species in Tunisia [1,8] and around the world [4], which promotes their mixing. Interestingly, in addition to the two major clades described corresponding to both rodent species *J. jaculus* and *J. hirtipes*, two phylogroups were clustered for each rodent species. To our knowledge, this is the first study to describe the presence of two well-supported phylogroups among *J*. *jaculus* and *J. hirtipes* in Tunisia and North Africa. The genetic distance evidence from *Cytb* sequences supports the coexistence of two phylogroups among these rodent species. In the same frame, there is no point in the ecosystem biotope for phylogroups of both rodent species, because they cohabit within the same zones. This confirms their sympatric speciation at micro-geographical and environmental scales, in addition to their sympatry at the macrogeographic scale previously described [4]. Reported worldwide climate changes of such magnitude are known to play a crucial role in shaping species distributions and may have triggered population expansion, as previously described and predicted in Iran for other species belonging to this genus: *J*. *blanfordi* [40].

### 4.4. Genetic Diversity, Polymorphism, and Differentiation among Rodent Species, Groups Belonging to Biotopes, and Phylogroups

According to standard genetic statistics, *J. jaculus* specimens from Guermessa appear to be more polymorphic than those from BniMhira, whereas the reverse image holds true for *J. hirtipes* specimens. This polymorphism can be partly explained by the scale of ecological divergence for each rodent species: *J. jaculus* and *J. hirtipes* were more adapted to the Saharan and mountainous biotopes, respectively. However, the small sample size of our study can lead to an overestimation [41]. These hypotheses should be addressed in future studies. Moreover, genetic diversity, genetic differentiation, and phylogenetic analyses were congruent, highlighting the existence of two well-supported clades (defined as *J. jaculus* and *J. hirtipes*), as recently described [4,10]. In addition, they highlight the clear separation into two phylogroups for each rodent species, despite the limited geographic area and small sampling size, which may underrepresent the genetic diversity at the intra-and inter-species levels. Large-scale sampling of these rodent species allows the conduction of morphometric, biochemical, genetic, cytogenetic, and essentially karyological studies that are now needed to confirm, without any suspicion, the presence of these different phylogroups that may represent another rodent species in Tunisia. This will also help update the geographical distribution of these species of rodents and identify their phylogroups. Therefore, the use of multiple other markers is needed to obtain a more reliable estimate of the phylogenetic diversification of this evolved species and its phylogroups. This will help to update the nomenclature of this rodent genus and resolve some of the problems related to the inclusion of many subspecies and other species (some of them belong to another genus) in the *Jaculus* genus [10]. 

## 5. Conclusions

This study confirms the phylotaxonomic status of Lesser Egyptian Jerboa in Tunisia, as recently illustrated, as two distinct species, *J. jaculus* and *J. hirtipes*. Indeed, the presence of two strongly supported phylogroups among these rodent species was recorded for the first time. Moreover, we determined sympatric speciation at the micro-geographic scale and highlighted the lack of biotope specification among these rodents at the species and phylogroup levels.

## Figures and Tables

**Table 1:** Morphological measurements and eye lens weight (ELW) of the captured rodents.

	Mean	SD	Minimum	Maximum	IQR
**Weight (g)**	42.52	9.42	25	65	35–50.5
**Head and body length (mm)**	108.13	8.89	90	128	100.75–115
**Tail length (mm)**	177.58	9.09	160	196	170–184.25
**Ear length (mm)**	21.36	1.58	18	24	20–22.25
**Hind feet length (mm)**	62.5	1.87	59	67	61–64
**ELW (mg)**	75.26	28.47	44	148	56.25–99.5

Abbreviations: SD, Standard Deviation; IQR, Interquartile Range.

**Table 2:** Morphological measurements and eye lense weight (ELW) among Tunisian *Jaculus* *jaculus* and *Jaculus hirtipes*.

	*J. jaculus*	*J. hirtipes*	p *
Number of specimens	21	20	
Mean of body measurements (±SD)			
Weight (g)	41.29 ± 9.20	44.60 ± 9.35	0.26
Head and body length (mm)	106.71 ± 7.77	109.10 ± 9.19	0.37
Tail length (mm)	176.38 ± 9.10	178.55 ± 9.04	0.449
Ear length (mm)	20.52 ± 1.40	22.25 ± 1.20	<10^−3^
Hind feet length (mm)	63.29 ± 1.79	62 ± 1.65	0.022
Mean of ELW (±SD) (mg)	79.19 ± 31.05	73.1 ± 27.28	0.509

Abbreviations: SD, Standard Deviation; *: Statistical significance was set at a value of *p* ≤ 0.0083 using the Bonferroni correction method.

**Table 3:** ELW associated with age categorization using the ELW-cutoff method.

	Number	Mean	SD	Minimum	Maximum
**Juveniles (mg)**	14	51.43	4.65	44	58
**Adults (mg)**	32	85.69	28.26	59	148

Abbreviation: SD, Standard Deviation.

**Table 4:** Standard genetic statistics of Tunisian *Jaculus* according to rodent species, biotopes, and phylogroups.

	*n*	H	Hd ± SD	S	π ± SD	K	Tajima’s D	Fu’s F_S_
**Sequences from the present study**								
	41	38	0.995 ± 0.007	141	0.071 ± 0.002	49.052	1.032	−6.648
**According to species identification**								
*J. jaculus*	21	20	0.995 ± 0.016	82	0.021 ± 0.002	17.347	−1.212	−6.445
*J. hirtipes*	20	18	0.984 ± 0.024	68	0.022 ± 0.002	17.273	−0.455	−4.038
**According to the biotope**								
**BNIMHIRA**								
*J. jaculus*	13	12	0.987 ± 0.035	62	0.020 ± 0.002	16.923	−0.866	−1.904
*J. hirtipes*	13	13	1 ± 0.03	67	0.026 ± 0.004	19.012	−0.597	−3.384
**GUERMESSA**								
*J. jaculus*	8	8	1 ± 0.063	54	0.022 ± 0.002	19.571	−0.506	−0.949
*J. hirtipes*	7	5	0.857 ± 0.137	37	0.018 ± 0.004	16.190	0.413	2.704
**Tunisian sequences retrieved from the GenBank**								
	41	35	0.988 ± 0.01	170	0.053 ± 0.004	48.559	0.691	−3.132
Whole Dataset								
	82	67	0.991 ± 0.005	182	0.065 ± 0.002	43.329	0.109	−14.946
**According to the species identification**								
*J. jaculus*	50	44	0.99 ± 0.008	151	0.022 ± 0.002	17.779	−1.893 *	−21.532
*J. hirtipes*	32	26	0.98 ± 0.015	75	0.022 ± 0.002	15.792	−0.653	−6.682
**According to the phylogroups**								
***J. jaculus***								
Phylogroup 1	9	8	0.972 ± 0.064	50	0.016 ± 0.002	15.388	−0.917	−0.190
Phylogroup 2	41	36	0.987 ± 0.012	123	0.018 ± 0.002	14.793	−1.990 *	−17.345
***J. hirtipes***								
Phylogroup 1	12	12	1 ± 0.034	51	0.016 ± 0.004	12.757	−1.190	−4.063
Phylogroup 2	20	15	0.963 ± 0.028	24	0.005 ± 0.0007	4.715	−1.168	−6.981

n, number of individuals; H, number of haplotypes; Hd, haplotype diversity; S, polymorphic sites; π, nucleotide diversity; K, average number of nucleotide differences. SD = standard deviation. *p* value (*) < 0.05.

**Table 5:** Genetic differentiation estimates for Tunisian *J. jaculus* and *J. hirtipes* and their phylogroups based on *Cytb* gene sequences comparison.

Comparison	Ks *	Kst *	Z *	*Snn*	*Fst*
*J. jaculus* vs. *J. hirtipes* Clade 1 vs. Clade 2	2.4471 ^a^	0.2716 ^a^	6.4423 ^a^	1 ^a^	0.812
*J. jaculus* phylogroups	2.5164 ^a^	0.0803 ^a^	5.8349 ^a^	1 ^a^	0.4618
Phylogroup 1 vs. Phylogroup 2					
*J. hirtipes* phylogroups	1.7876 ^a^	0.2762 ^a^	4.6813 ^a^	1 ^a^	0.6961
Phylogroup 1 vs. Phylogroup 2					

^a^: *p*-value < 0.001; Ks *, Kst *, Z *, and *Snn*: test statistics of genetic differentiation; *Fst* is the coefficient of gene differentiation, which measures the inter-population diversity.

## Data Availability

*Cytb* gene sequences were submitted to the GenBank database under accession numbers: OL898609–OL898649. Addresses are as follows: www.ncbi.nlm.nih.gov/genbank accessed on 8 March 2022.

## Supplementary Materials

The following supporting information can be downloaded at: https://www.mdpi.com/article/10.3390/ani12060758/s1, Table S1: Sequences of Cytochrome b of rodents belonging to J. jaculus and J. hirtipes used in the phylogenetic analysis.

## References

[1] Ben Faleh A., Cosson J.F., Tatard C., Ben Othmen A., Said K., Granjon L. (2010). Are there two cryptic species of the lesser jerboa *Jaculus jaculus* (Rodentia: Dipodidae) in Tunisia? Evidence from molecular, morphometric, and cytogenetic data. Biol. J. Linn. Soc..

[2] Boratyński Z., Brito J.C., Campos J.C., Karala M., Mappes T. (2014). Large spatial scale of the phenotype-environment color matching in two cryptic species of African desert jerboas (Dipodidae: *Jaculus*). PLoS ONE.

[3] Ranck G.L. (1968). The rodents of Libya: Taxonomy, ecology, and zoogeographical relationships. Bull. US Natl. Mus..

[4] Moutinho A.F., Seren N., Pauperio J., Silva T.L., Martinez-Freiria F., Sotelo G., Faria R., Mappes T., Alves P.C., Brito J.C. (2020). Evolutionary history of two cryptic species of northern African jerboas. BMC Evol. Biol..

[5] Ben Faleh A., Ben Othmen A., Said K. (2010). Taxonomy of the lesser jerboa *Jaculus jaculus* (Dipodidae, Rodentia) based on allozymic and morphological variation. Curr. Zool..

[6] Ben Faleh A., Ben Othmen A., Said K., Granjon L. (2010). Karyotypic variation in two species of jerboas *Jaculus jaculus* and *Jaculus orientalis* (Rodentia, Dipodidae) from Tunisia. Folia Biol..

[7] Ben Faleh A., Cornette R., Annabi A., Said K., Denys C. (2013). Patterns of size and shape skull variability in Tunisian populations of *Jaculus jaculus* (Rodentia: Dipodidae). Acta Zool. Bulg..

[8] Ben Faleh A., Granjon L., Tatard C., Boratynski Z., Cosson J.F., Said K. (2012). Phylogeography of two cryptic species of African desert jerboas (Dipodidae: *Jaculus*). Biol. J. Linn. Soc..

[9] Boratynski Z., Brito J.C., Mappes T. (2012). The origin of two cryptic species of African desert jerboas (Dipodidae: *Jaculus*). Biol. J. Linn. Soc..

[10] Shenbrot G., Feldstein T., Meiri S. (2016). Are cryptic species of the Lesser Egyptian Jerboa, *Jaculus jaculus* (Rodentia, Dipodidae), really cryptic? Re-evaluation of their taxonomic status with new data from Israel and Sinai. J. Zool. Syst. Evol. Res..

[11] Shahin A.A.B. (2003). Genetic differentiation and relationship of the dipodids *Allactaga* and *Jaculus* (Mammalia, Rodentia) in Egypt based on protein variation. Acta Theriol..

[12] Ben Faleh A., Granjon L., Tatard C., Cosson J.F., Said K. (2013). Phylogeography of two cryptic species of African desert jerboas (Dipodidae: *Jaculus*). Biol. J. Linn. Soc..

[13] Ben Faleh A., Shahin A.A., Said K. (2009). Allozyme polymorphism and genetic differentiation among populations of *Jaculus jaculus* and *J. orientalis* (Rodentia: Dipodidae) in Tunisia. Zool. Res..

[14] Eymann J., Degreef J., Häuser C., Monje J., Samyn Y., VandenSpiegel D. (2010). Manual on Field Recording Techniques and Protocols for All Taxa Biodiversity Inventories and Monitoring.

[15] Jacques M.-E., McBee K., Elmore D. (2015). Determining Sex and Reproductive Status of Rodents.

[16] Hadjoudj M., Manaa A., Derdoukh W., Guerzou A., Souttou K., Sekour M., Doumandji S. (2011). Les Rongeurs de la Région de Touggourt.

[17] Fichet-Calvet E., Jomaa I., Ben Ismail R., Ashford R.W. (2003). *Leishmania major* infection in the fat sand rat *Psammomys obesus* in Tunisia: Interaction of host and parasite populations. Ann. Trop. Med. Parasitol..

[18] Granjon L., Duplantier J.-M. (2009). Les Rongeurs de l’Afrique Sahélo-Soudanienne.

[19] Morris P. (1972). A review of mammalian age determination methods. Mamm. Rev..

[20] Higgins D.G., Thompson J.D., Gibson T.J. (1996). Using CLUSTAL for multiple sequence alignments. Methods Enzymol..

[21] Kumar S., Nei M., Dudley J., Tamura K. (2008). MEGA: A biologist-centric software for evolutionary analysis of DNA and protein sequences. Brief. Bioinform..

[22] Gouy M., Guindon S., Gascuel O. (2010). SeaView version 4: A multiplatform graphical user interface for sequence alignment and phylogenetic tree building. Mol. Biol. Evol..

[23] Nei M., Tajima F. (1987). Problems Arising in Phylogenetic Inference from Restriction-Site Data. Mol. Biol. Evol..

[24] Tajima F. (1989). Statistical method for testing the neutral mutation hypothesis by DNA polymorphism. Genetics.

[25] Fu B., Curry F.R., Adamson R.H., Weinbaum S. (1997). A model for interpreting the tracer labeling of interendothelial clefts. Ann. Biomed. Eng..

[26] Rozas J., Ferrer-Mata A., Sanchez-DelBarrio J.C., Guirao-Rico S., Librado P., Ramos-Onsins S.E., Sanchez-Gracia A. (2017). DnaSP 6: DNA sequence polymorphism analysis of large data sets. Mol. Biol. Evol..

[27] Hudson R.R. (2000). A new statistic for detecting genetic differentiation. Genetics.

[28] Hudson R.R., Boos D.D., Kaplan N.L. (1992). A statistical test for detecting geographic subdivision. Mol. Biol. Evol..

[29] Tsompana M., Abad J., Purugganan M., Moyer J.W. (2005). The molecular population genetics of the Tomato spotted wilt virus (TSWV) genome. Mol. Ecol..

[30] Gao F., Lin W., Shen J., Liao F. (2016). Genetic diversity and molecular evolution of arabis mosaic virus based on the CP gene sequence. Arch. Virol..

[31] Ben Faleh A., Allaya H., Quignard J.P., Shahin A.A.A.B., Trabelsi M. (2016). Patterns of skull variation in relation to some geoclimatic conditions in the greater jerboa *Jaculus orientalis* (Rodentia, Dipodidae) from Tunisia. Turkish J. Zool..

[32] Ben Faleh A., Allaya H., Shahin A.A.A.B. (2016). Geographic patterns of genetic variation in the greater Egyptian jerboa *Jaculus orientalis* (Dipodidae, Rodentia) from Tunisia. Biochem. Syst. Ecol..

[33] Happold D. (1967). Biology of the jerboa, *Jaculus jaculus butleri* (Rodentia, Dipodidae), in the Sudan. J. Zool..

[34] Maatoug H. (2018). Etude Morpho-Métrique des Rongeurs dans la Région du Souf. Master’s Thesis.

[35] Ghawar W., Snoussi M.-A., Salem S., Jaouadi K., Zaatour W., Yazidi R., Bettaieb J. (2017). Morphometric variation and its relation to the eyes lens weight among three species of wild rodents in Tunisia. Int. J. Fauna Biol. Stud..

[36] Lalis A., Evin A., Janier M., Koivogui L., Denys C. (2015). Host evolution in *Mastomys natalensis* (Rodentia: Muridae): An integrative approach using geometric morphometrics and genetics. Integr. Zool..

[37] Gharaibeh B.M. (1997). Systematics, Distribution, and Zoogeography of Mammals of Tunisia.

[38] Pisano J., Condamine F.L., Lebedev V., Bannikova A., Quere J.P., Shenbrot G.I., Pages M., Michaux J.R. (2015). Out of Himalaya: The impact of past Asian environmental changes on the evolutionary and biogeographical history of Dipodoidea (Rodentia). J. Biogeogr..

[39] Corbet G.B. (1978). The Mammals of the Palaearctic Region: A Taxonomic Review.

[40] Mohammadi S., Ebrahimi E., Moghadam M.S., Bosso L. (2019). Modelling current and future potential distributions of two desert jerboas under climate change in Iran. Ecol. Inform..

[41] Stumpf M.P. (2004). Haplotype diversity and SNP frequency dependence in the description of genetic variation. Eur. J. Hum. Genet..

