# Supplementary material for: Evaluation of the Taxonomic Status of Lesser Egyptian Jerboa, Jaculus jaculus: First Description of New Phylogroups in Tunisia
Source: Animals (Basel). 2022 Mar 17;12(6):758. doi: 10.3390/ani12060758 (PMC8944451; doi:10.3390/ani12060758)
Supplement: Supplementary file 1 [file animals-12-00758-s001.zip › animals-1548445-supplementary.pdf]

Table S1: Sequences of Cytochrome b of rodents belonging to *J. jaculus* and *J. hirtipes* used in the phylogenetic analysis.

Specimen	Identified Species	Region	Governorate	Country	Genebank ID
Jac 1	*J. jaculus*	Guermessa	Tataouine	Tunisia	OL898641
Jac 2	*J. jaculus*	Guermessa	Tataouine	Tunisia	OL898644
Jac 3	*J. jaculus*	Guermessa	Tataouine	Tunisia	OL898645
Jac 4	*J. hirtipes*	BniMhira	Tataouine	Tunisia	OL898613
Jac 5	*J. hirtipes*	BniMhira	Tataouine	Tunisia	OL898628
Jac 6	*J. jaculus*	BniMhira	Tataouine	Tunisia	OL898642
Jac 7	*J. hirtipes*	BniMhira	Tataouine	Tunisia	OL898626
Jac 8	*J. hirtipes*	BniMhira	Tataouine	Tunisia	OL898627
Jac 9	*J. hirtipes*	BniMhira	Tataouine	Tunisia	OL898624
Jac 10	*J. jaculus*	BniMhira	Tataouine	Tunisia	OL898634
Jac 11	*J. hirtipes*	BniMhira	Tataouine	Tunisia	OL898616
Jac 12	*J. jaculus*	BniMhira	Tataouine	Tunisia	OL898643
Jac 13	*J. hirtipes*	BniMhira	Tataouine	Tunisia	OL898625
Jac 14	*J. hirtipes*	BniMhira	Tataouine	Tunisia	OL898621
Jac 15	*J. jaculus*	BniMhira	Tataouine	Tunisia	OL898648
Jac 16	*J. jaculus*	BniMhira	Tataouine	Tunisia	OL898647
Jac 17	*J. hirtipes*	BniMhira	Tataouine	Tunisia	OL898617
Jac 19	*J. jaculus*	BniMhira	Tataouine	Tunisia	OL898646
Jac 20	*J. jaculus*	BniMhira	Tataouine	Tunisia	OL898631
Jac 21	*J. jaculus*	BniMhira	Tataouine	Tunisia	OL898632
Jac 23	*J. hirtipes*	BniMhira	Tataouine	Tunisia	OL898615
Jac 25	*J. hirtipes*	BniMhira	Tataouine	Tunisia	OL898619
Jac 27	*J. hirtipes*	BniMhira	Tataouine	Tunisia	OL898614
Jac 28	*J. jaculus*	BniMhira	Tataouine	Tunisia	OL898649
Jac 29	*J. jaculus*	BniMhira	Tataouine	Tunisia	OL898633
Jac 30	*J. jaculus*	BniMhira	Tataouine	Tunisia	OL898635
Jac 31	*J. jaculus*	BniMhira	Tataouine	Tunisia	OL898636
Jac 32	*J. hirtipes*	BniMhira	Tataouine	Tunisia	OL898622
Jac 33	*J. jaculus*	BniMhira	Tataouine	Tunisia	OL898640
Jac 34	*J. hirtipes*	Guermessa	Tataouine	Tunisia	OL898623
Jac 35	*J. hirtipes*	Guermessa	Tataouine	Tunisia	OL898620
Jac 36	*J. jaculus*	Guermessa	Tataouine	Tunisia	OL898637
Jac 37	*J. jaculus*	Guermessa	Tataouine	Tunisia	OL898639
Jac 38	*J. jaculus*	Guermessa	Tataouine	Tunisia	OL898638
Jac 39	*J. hirtipes*	Guermessa	Tataouine	Tunisia	OL898618
Jac 40	*J. hirtipes*	Guermessa	Tataouine	Tunisia	OL898612
Jac 41	*J. hirtipes*	Guermessa	Tataouine	Tunisia	OL898611
Jac 43	*J. hirtipes*	Guermessa	Tataouine	Tunisia	OL898610
Jac 44	*J. hirtipes*	Guermessa	Tataouine	Tunisia	OL898609
Jac 45	*J. jaculus*	Guermessa	Tataouine	Tunisia	OL898630

Jac 46	*J. jaculus*	Guermessa	Tataouine	Tunisia	OL898629
		**References sequences**			
**Tunisian database from GeneBank**	*J. jaculus*	Douz	Tozeur	Tunisia	GU433412
	J. jaculus	Douz	Tozeur	Tunisia	GU433413
	J. jaculus	Douz	Tozeur	Tunisia	GU433424
	J. jaculus	Gabes	Gabes	Tunisia	GU433431
	J. jaculus	Hamma	Gabes	Tunisia	GU433414
	J. jaculus	Hamma	Gabes	Tunisia	GU433415
	J. jaculus	Hamma	Gabes	Tunisia	JX885166
	J. hirtipes	Matmata	Gabes	Tunisia	GU433435
	J. hirtipes	Matmata	Gabes	Tunisia	GU433436
	J. hirtipes	Matmata	Gabes	Tunisia	GU433438
	J. hirtipes	Matmata	Gabes	Tunisia	GU433440
	J. hirtipes	Matmata	Gabes	Tunisia	GU433441
	J. hirtipes	Matmata	Gabes	Tunisia	JX885168
	J. hirtipes	Matmata	Gabes	Tunisia	JX885169
	J. jaculus	Matmata	Gabes	Tunisia	GU433411
	J. jaculus	Matmata	Gabes	Tunisia	GU433416
	J. jaculus	Matmata	Gabes	Tunisia	GU433417
	J. jaculus	Matmata	Gabes	Tunisia	GU433418
	J. jaculus	Matmata	Gabes	Tunisia	GU433420
	J. jaculus	Matmata	Gabes	Tunisia	GU433421
	J. jaculus	Matmata	Gabes	Tunisia	GU433425
	J. jaculus	Matmata	Gabes	Tunisia	GU433426
	J. jaculus	Matmata	Gabes	Tunisia	GU433427
	J. jaculus	Matmata	Gabes	Tunisia	GU433428
	J. jaculus	Matmata	Gabes	Tunisia	GU433429
	J. jaculus	Matmata	Gabes	Tunisia	GU433430
	J. jaculus	Matmata	Gabes	Tunisia	JX885167
	J. hirtipes	Medenine	Medenine	Tunisia	JX885170
	J. hirtipes	Menzel Chaker	Sfax	Tunisia	GU433433
	J. jaculus	Menzel Chaker	Sfax	Tunisia	GU433422
	J. jaculus	Nefta	Tozeur	Tunisia	GU433407
	J. jaculus	Nefta	Tozeur	Tunisia	GU433408
	J. jaculus	Nefta	Tozeur	Tunisia	GU433409
	J. jaculus	Nefta	Tozeur	Tunisia	GU433410
	J. jaculus	Nefta	Tozeur	Tunisia	GU433419
	J. jaculus	Remada	Tataouine	Tunisia	GU433432
	J. hirtipes	Sbeitla	Kasserine	Tunisia	GU433434
	J. hirtipes	Sbeitla	Kasserine	Tunisia	GU433437
	J. hirtipes	Sbeitla	Kasserine	Tunisia	GU433439
	J. jaculus	Tataouine	Tataouine	Tunisia	GU433423
	J. jaculus	Tataouine	Tataouine	Tunisia	JN214516
**Out group**	*J. orientalis*	Maktheur	Siliana	Tunisia	JN652641
	Psammomys vexillaris	Kebili	Kebili	Tunisia	MF687143
